# Transcriptional Induction of NF-kB-Inducing Kinase by E2F4/5 Facilitates Collective Invasion of Glioma Cells

**DOI:** 10.21203/rs.3.rs-2622363/v1

**Published:** 2023-03-07

**Authors:** Kathryn Pflug, Dong Lee, Kassandra McFadden, Linda Herrera, Raquel Sitcheran

**Affiliations:** Texas A&M Health Science Center; Texas A&M Health Science Center; Texas A&M Health Science Center; Texas A&M Health Science Center; Texas A&M Health Science Center

## Abstract

The prognosis of high-grade gliomas, such as glioblastoma multiforme (GBM), is extremely poor due to the highly invasive nature of these aggressive cancers. Previous work has demonstrated that TNF-weak like factor (TWEAK) induction of the noncanonical NF-κB pathway increases the invasiveness of glioma cells in an NF-κB-inducing kinase (NIK)-dependent manner. While NIK activity is predominantly regulated at the posttranslational level, we show here that NIK (*MAP3K14*) is upregulated at the transcriptional level in invading cell populations, with the highest expression observed in the most invasive cells. Glioma cells with high induction of NIK gene expression demonstrate characteristics of collective invasion, facilitating invasion of neighboring cells. Furthermore, we demonstrate that the E2F transcription factors E2F4 and E2F5 directly regulate NIK transcription and are required to promote glioma cell invasion in response to TWEAK. Overall, our findings demonstrate that transcriptional induction of NIK facilitates collective cell migration and invasion, thereby promoting glioma pathogenesis.

## Introduction

While glioblastoma multiforme (GBM) tumors rarely metastasize outside of the central nervous system (CNS), their aggressive growth and persistent invasiveness into healthy brain tissue are major factors underlying resistance to conventional treatment methods such as surgery, irradiation, and chemotherapy^1,2^. In addition to single-cell invasion, multicellular, connected networks of glioma cells have been observed in human tumors and mouse models^3–5^. This collective invasion, defined by the coordinated movement of cells into surrounding tissue while maintaining cell-cell junctions, is a process that occurs in epithelial regeneration and during the development and remodeling of large tissue structures, including angiogenesis and neural crest cell streaming. Collective invasion has also been identified as a significant mode of invasion in glioma^6,7^. During collective invasion, cells have been found to acquire leader-follower phenotypes, in which both leader (pioneer) and follower cells assume different metabolic activities and work in concert to promote tumor cell dispersion. Leader cells direct the leading edge of the tumor, migrating through the microenvironment, paving the path of invasion, and transmitting information to follower cells^8,9^. However, there is an increasing body of evidence that tumor cells in collective invasion may alternate between leader and follower phenotypes, warranting further studies of this process.

Activation of NF-κB signaling pathways has been well documented in a variety of malignancies, including GBM^10^. We previously demonstrated that activation of the noncanonical NF-κB pathway through TNF-weak-like factor (TWEAK) is a potent inducer of GBM invasion^11^. Specifically, NF-κB-inducing kinase (NIK; encoded by *MAP3K14*), which is an essential upstream kinase for noncanonical NF-κB activation, increases matrix metalloproteinase 1 (MT1-MMP) activity to promote invadopodia formation during glioma invasion^12^. NIK is generally described as being regulated at the posttranslational level, whereby constitutive proteosome-dependent NIK protein turnover is attenuated in a signal-dependent manner, resulting in the accumulation of catalytically active NIK. In the current study, we show that the dynamic, signal-dependent transcriptional upregulation of NIK is pronounced in cells at the leading edge of gliomaspheres, enhancing collective invasion of neighboring cells into the surrounding matrix.

## Results

### Induction of NIK Transcription Directly Correlates with Invasion

**Figure 1:**
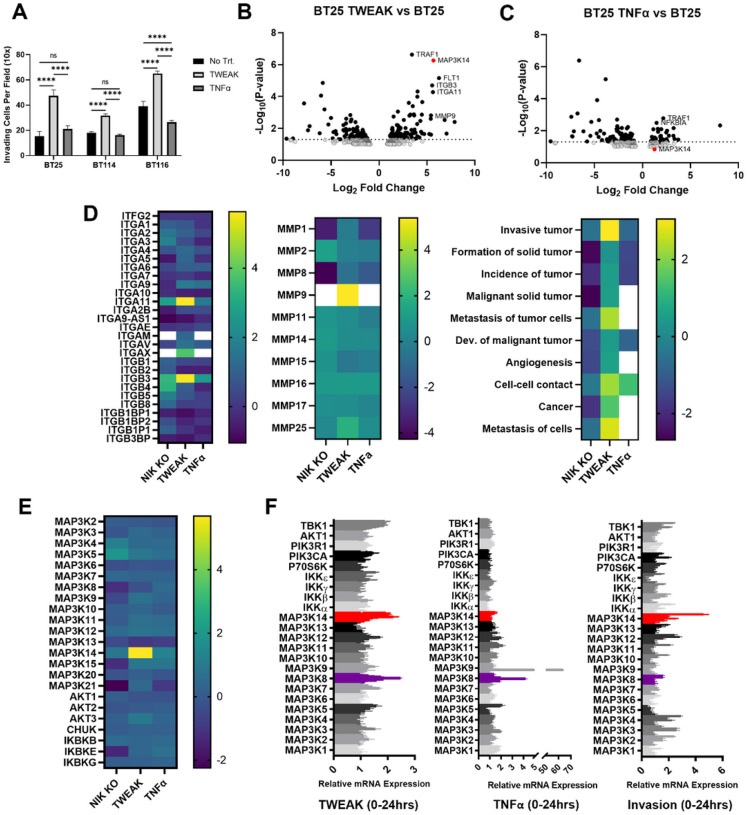
Specific induction of NIK promotes invasion **A)** Quantification of three-dimensional collagen invasion assay BT25, BT114, and BT116 GBM cells after 48 hours. Invasion was conducted under basal, TWEAK treatment (10 ng/mL), or TNFα (10 ng/mL). Statistical analysis was performed using two-way ANOVA p < 0.001. **B-E)** RNA sequencing analysis of BT25 GBM cells with either TWEAK treatment (10 ng/mL for 4 hr) or TNFα treatment (10 ng/mL for 30 minutes) on wild-type cells or NIK KO BT25 cells compared to wild-type BT25 cell gene expression. IPA software was used for pathway analyses. **F)** RT-qPCR analysis was utilized to further examine the expression of NF-κB proteins and MAP3Ks in BT114 GBM cells. Cells were treated with either 10 ng/mL TWEAK or 10 ng/mL TNFα at 0, 0.25, 0.5, 1, 4, 8, 16, and 24 hours. RT-qPCR analysis of cells extracted from the collagen matrix at 0, 0.5, 4, 8, and 24 hours post invasion.

Consistent with our previous findings^11^, we observed that the invasiveness of the human-derived glioblastoma (GBM) cell lines BT25, BT114, and BT116 was significantly induced by treatment with TWEAK, whereas treatment with TNFα did not stimulate invasion (Figure 1A). Transcriptome analysis of BT25 cells treated with TWEAK or TNFα, which preferentially activate the canonical or noncanonical NF-κB pathways, respectively^13,14^, revealed that the expression of NIK (*MAP3K14*) directly correlated with glioma cell invasion and was highly induced in response to TWEAK treatment but not TNFα treatment (Figure 1B, C). Moreover, we observed TWEAK-specific upregulation of tumor necrosis factor receptor (TNFR)-associated factor 1 (TRAF1), a signaling adapter that interacts with and stabilizes NIK for activation of the noncanonical NF-κB pathway^15^. Elevated levels of integrin β3 (ITGB3), integrin subunit alpha 11 (ITGA11), matrix metalloproteinase 9 (MMP9), and Fms-related tyrosine kinase (FLT1) were also observed, consistent with increased invasive and migratory potential^16^. In addition to the induction of ITGB3, we observed that TWEAK treatment also elevated the expression of ITGA11, which has previously been described in glioma invasion^17^. Furthermore, only TWEAK-treated glioma cells exhibited increased expression of integrins, as well as MMP9, all of which are associated cancer markers^18–21^. Ingenuity Pathway Analysis (IPA) of groups of genes belonging to specific diseases and functions revealed that TWEAK treatment elevated the overall expression of cancer pathways, including tumor formation, invasion, and metastasis (Figure 1D). Additionally, RNA-seq analysis demonstrated that relative to other MAP3 kinases and upstream regulatory kinases in the NF-κB signaling pathways, TWEAK treatment singularly induced NIK/*MAP3K14* gene expression (Figure 1E). For example, while qPCR analysis showed high expression of *MAP3K14* as well as *MAP3K8* in TWEAK-treated cells, *MAP3K8* was also induced by both TNF and TWEAK. Moreover, analysis of cells actively undergoing invasion in collagen matrices revealed that NIK/*MAP3K14* was the most highly upregulated gene compared to other kinases (Figure 1F). We also observed TWEAK-induction of NIK mRNA and invasion in other GBM cell lines, as well as mouse embryonic broblasts (MEFs) (Supp. Figure 1A, B, C).

After observing elevated NIK transcription and invasion upon TWEAK treatment, we investigated potential paracrine effects among glioma cells during invasion. We found that BT116 cells underwent increased invasion when treated with conditioned media (Control CM) from highly invasive BT25 cells compared with unconditioned media. This increased invasion was dependent on NIK, as it was not observed when BT116 cells were cultured with conditioned media from NIK knockout BT25 cells (NIK KO CM), while conditioned media from NIK KO cells rescued with ectopic expression of murine NIK (NIK KO-mNIK CM) restored the increased invasion (Supp. Figure 1D-E). Consistent with the results from media treatments, direct coculture of BT25 and BT114 cells increased the cell invasion/migration of the BT114 cells compared to cells cultured alone (Supp. Figure 1F). These results demonstrate that transcriptional induction of NIK is associated with elevated glioma invasiveness that is propagated by NIK-dependent paracrine signaling.

### NIK Expression is Upregulated in Highly Invasive Glioma Cells and Promotes Collective Invasion

**Figure 2:**
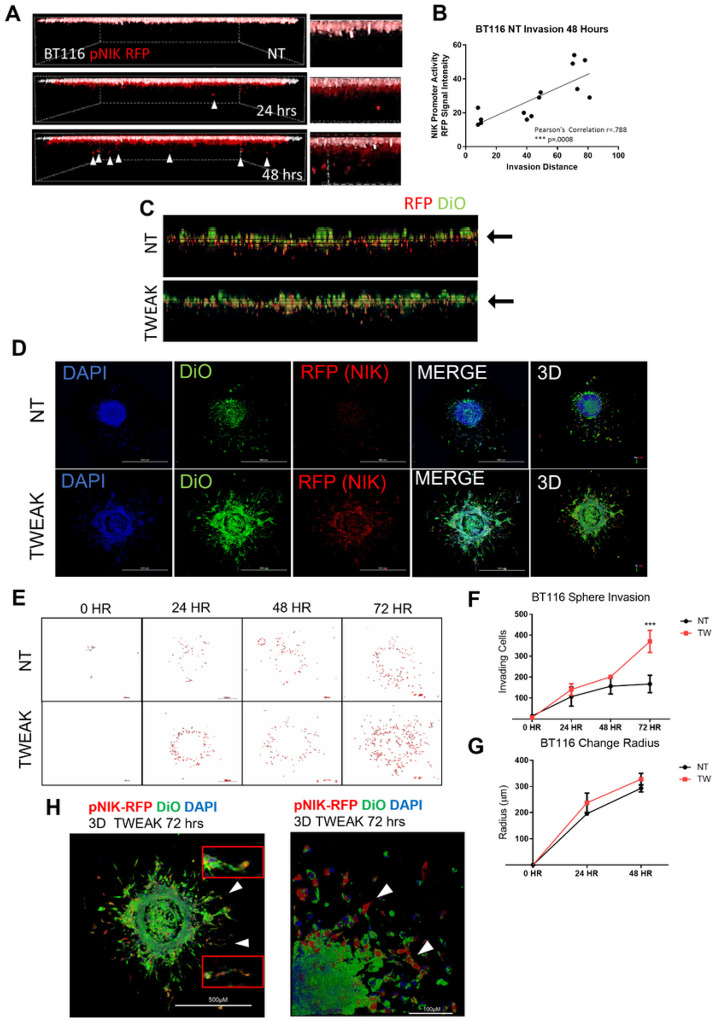
NIK Expression is Upregulated in Highly Invasive Glioma Cells and Promotes Collective Invasion **A)** Live-cell confocal microscopy was utilized to visualize the RFP reporter under the NIK promoter (pNIK-RFP) (red) under unstimulated conditions (NT). The monolayer (0 hrs) was pseudocolored white and overlaid to the reference invasion distance. **B)** Graph representation of RFP intensity after 48 hours and distance the cells invaded. Pearson’s correlation between RFP intensity and distance is r=.788 and p=.008, ***p≤.001. **C)** Invasion of BT116 pNIK-RFP cells labeled with DiO (green) with no treatment (NT) or treated with 10 ng/mL TWEAK. **D)** BT116 pNIK-RFP cells were used for a spheroid invasion assay. Spheroids were embedded in three-dimensional collagen matrix and either left untreated (NT) or treated with TWEAK (10 ng/mL). Spheres were allowed to invade for 72 hours. Confocal microscopy was used to image spheroids, labeled DiO (green), RFP reporter (red), and DAPI (blue). **E)** Outline of BT116 pNIK-RFP (red) spheroids embedded in three-dimensional collagen for 0–72 hr, either untreated or treated with TWEAK (TW) for 48 hours. **F)** The ImageJ particle analysis function was used to quantify the number of cells invading into the three-dimensional collagen matrix at various time points. **G)** ImageJ was used to measure the radius of the spheroids at various time points. **H)** Three-dimensional projection image BT116 pNIK-RFP (Red) spheroids were embedded in three-dimensional collagen and treated with TWEAK (TW) for 72 hours. Cells were labeled with DiO (green) and DAPI (blue). White **arrowheads** with enhanced images of cells undergoing collective invasion. Spheroid invasion assays were conducted in n>3 either untreated or treated with 10 ng/mL TWEAK.

To monitor the induction of NIK transcription during invasion *in vivo*, we generated BT116 glioma cells stably expressing red fluorescent protein (RFP) under the promoter of NIK (pNIK-RFP). Analysis of invading BT116 pNIK-RFP cells revealed a general induction of NIK expression in invading cells (red signal from monolayer pseudocolored white) (Figure 2A), with the farthest invading cells exhibiting the highest RFP intensity or highest expression of NIK (Figure 2B). Treatment of BT116 pNIK-RFP cells with TWEAK further increased NIK expression (RFP signal-red/yellow), with RFP-positive cells invading farther under either untreated (NT) or TWEAK-treated conditions (Figure 2C).

To evaluate collective invasion and leader-follower phenotypes in a 3-dimensional view, we performed invasion assays of glioma tumor spheres, which better mimic cell-cell and cell-matrix interactions. Imaging of collagen-embedded BT116 pNIK-RFP spheres revealed an increase in pNIK-RFP expression upon TWEAK treatment (Figure 2D), with the highest pNIK-RFP expression observed among the most invasive cells at the leading edge of the sphere (Supp. Fig 2A, Figure 2D, E). Consistent with single-cell invasion assays, the induction of NIK expression directly correlated with the migration and dispersion of cells from glioma spheres, which was enhanced upon TWEAK treatment (Figure 2F) compared to the invasion of similar-sized spheres from the untreated group (Figure 2G). Cells with a high pNIK-RFP signal were observed among cells undergoing collective invasion at the sphere peripheries (Figure 2H). These results demonstrate that cells with the highest TWEAK-induced NIK expression directly correlated with the most invasive glioma cells, facilitating collective invasion, consistent with increased invasion and metastasis gene signatures.

### Inhibition of NIK Activity Reduces Glioma Cell Invasion

**Figure 3:**
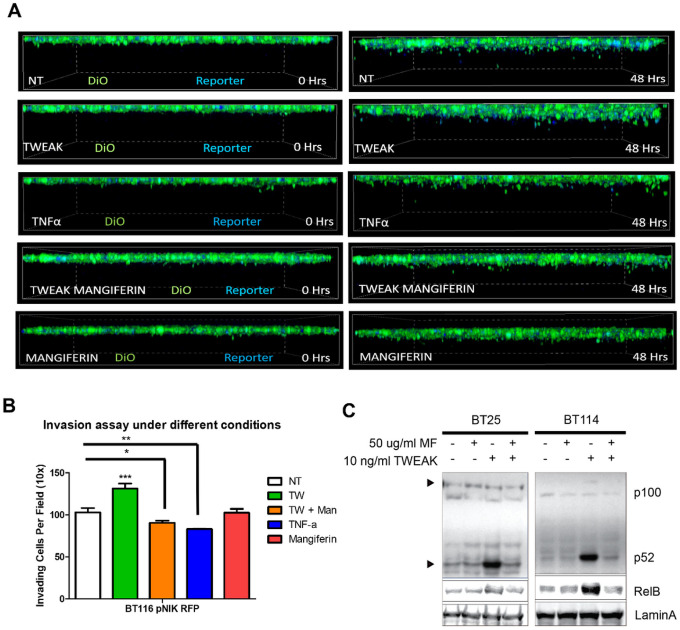
Inhibition of NIK Reduces Glioma Invasion **A)** BT116 pNIK RFP cells were labeled with DiO before being seeded on collagen matrix and allowed to invade for 48 hours. Live cell confocal microscopy was used to obtain Z-stack images over 48 hours, with the top layer depicting the monolayer at 0 hr and the bottom image depicting cell invasion at 48 hr. Cells either received no treatment (NT) or were treated with 10 ng/mL TWEAK,10 ng/mL TNF-α, 10 ng/ml TWEAK with 100 μg/mL mangiferin, or 100 μg/mL mangiferin. **B)** Quantification of cell invasion after 48 hours. **C)** Immunoblot showing decreased RelB and p52 in the nucleic fraction of glioma cells treated with mangiferin and/or TWEAK.

Next, we evaluated whether NIK catalytic activity was required to promote GBM invasion. Treatment of cells with mangiferin, a natural inhibitor of NIK^22–24^, significantly attenuated TWEAK-stimulated invasion to levels comparable with untreated or TNF-treated conditions (Figure 3A, B). Mangiferin was verified to inhibit activation of the noncanonical NF-κB pathway by TWEAK treatment in glioma cells, as seen with a reduction in p100-p52 processing and RelB (Figure 3C). Mangiferin also inhibited TWEAK-induced NIK transcription (Supp. Fig. 2B). Although mangiferin inhibited NIK and the noncanonical NF-κB pathway, it did not affect cell proliferation (Supp. Figure 2C). These data suggest therapeutic potential of inhibiting NIK to attenuate glioma invasion.

### E2F4 and E2F5 Regulate NIK Gene Expression

**Figure 4:**
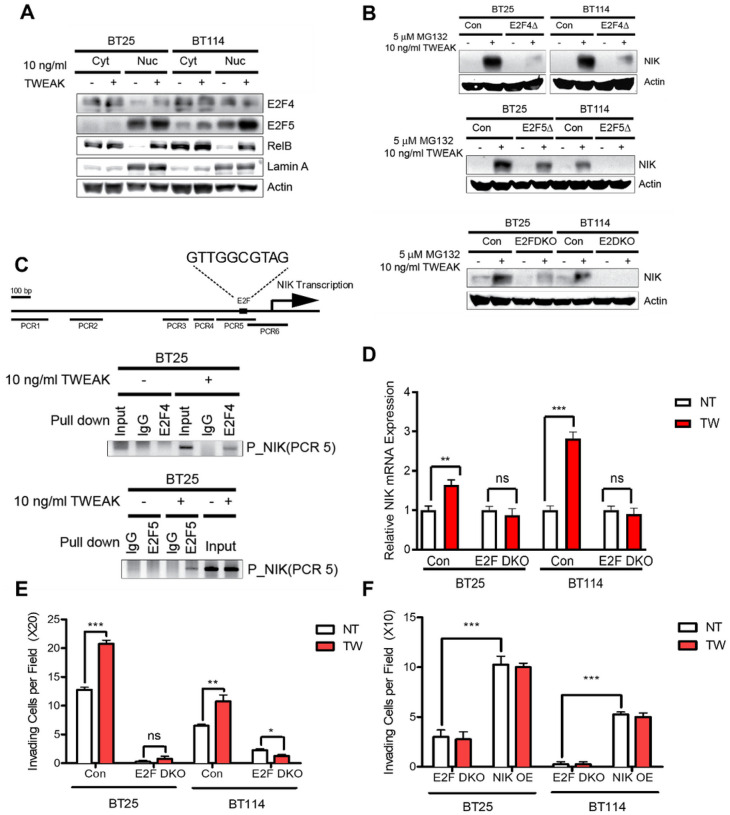
E2F4 and E2F5 Regulate NIK Gene Expression A) Cytoplasmic and nuclear E2F4 and E2F5 were examined by Western blot in unstimulated vs stimulated conditions (10 ng/mL TWEAK for 4 hrs) with RelB as a positive TWEAK-inducible control and Lamin A as a nuclear marker. **B)** Immunoblot of NIK expression under MG132 (5 μM)- and TWEAK (10 ng/mL)-stimulated conditions in control and E2F4, E2F5, or E2F4 and E2F5 knockout cells. **C)** ChIP analysis performed in glioma cells treated with or without 10 ng/mL TWEAK by using E2F4 or E2F5 antibodies. **D)** qPCR analysis of NIK (*MAP3K14*) induction in double knockout E2F4 and E2F5 glioma cells (BT25 and BT114). **E)** Invasion assay of BT25 and BT114 double knockout E2F4/E2F5 cells. **F)** Invasion assay of BT25 and BT114 double knockout E2F4 and E2F5 cells rescued by NIK overexpression. **D-F)** Comparisons were made using glioma cells in untreated (NT) vs TWEAK-treated (10 ng/mL for 4 hrs) conditions.

The mechanisms underlying the regulation of NIK gene expression are not fully understood. A recent study identified *MAP3K14* (NIK) as a significantly upregulated gene in human osteosarcoma cells with E2F activation^25^, and analysis of the NIK promoter revealed the presence of E2F binding sites (Supp. Fig. 3D). Thus, we next investigated whether E2F transcription factors played a role in early NIK gene expression in response to TWEAK treatment in glioma cells. We observed that TWEAK treatment increased nuclear E2F4 and E2F5 protein levels in glioma cells, with E2F4 having greater nuclear translocation in BT25 cells and E2F5 in BT114 cells (Figure 4A). We also found that overexpression of E2F5, but not E2F1, increased NIK transcript levels (Supp. Figure 3A). E2F4 and E2F5 single and double knockout cell lines generated by CRISPR-Cas9 gene editing exhibited a significant reduction in NIK protein levels when treated with TWEAK and MG132 to inhibit proteosome-dependent degradation (Supp. Figure 3B, Figure 4B). Moreover, E2F4/5 double knockout cells (E2F DKO) exhibited reduced p52/RelB nuclear translocation, demonstrating impaired activation of NIK-driven noncanonical NF-κB signaling (Supp. Figure 3C).

Next, we investigated whether E2F proteins directly regulate NIK transcription. Chromatin immunoprecipitation (ChIP) analyses demonstrated that antibodies specific to E2F4 and E2F5, but not IgG, were bound to certain regions of the NIK promoter (Figure 4C). Additionally, qPCR analysis of E2F-DKO cells showed significantly attenuated induction of NIK mRNA expression even with TWEAK treatment (Figure 4D). Reduced NIK expression in E2F DKO cells proved to have functional consequences, as these cells were poorly invasive, even after TWEAK treatment (Figure 4E), which was restored with ectopic expression of NIK (NIK OE) in E2F DKO cells (Figure 4F). Overall, these data establish a novel role for E2F regulation of NIK transcription in a stimulated state and thus affect the cell’s overall ability to invade (Supp. Fig. 3F).

## Discussion

While posttranscriptional regulation of NIK protein stability is important for controlling the activation of NF-κB signaling, in this study, we report that signal-specific transcriptional upregulation of NIK, but not other related kinases, is strongly associated with enhanced collective invasion of glioma cells(Figure 1). RNA-seq and qPCR analyses also verified that TWEAK but not TNFα treatment elevated NIK transcription, which was also observed in actively invading glioma cells. Using a NIK promoter-controlled reporter construct as a readout for NIK gene expression levels, we demonstrate that TWEAK-induced NIK transcription directly correlates with cell invasion and the acquisition of leader-follower cells seen in collective invasion (Figure 2H). Indeed, we observed that the farthest invading cells expressed the highest levels of NIK transcription (Figure 2). Our data showing that conditioned media from NIK KO glioma cells was unable to stimulate invasion, while conditioned media from NIK KO-mNIK cells rescued invasion, suggests that NIK-dependent paracrine signaling propagates a collective leader-follower cell phenotype during cell invasion. Furthermore, although prior studies have shown that E2F regulation of cIAPs and E2F1 directly promotes *MAP3K14* gene expression in osteosarcoma cells^25–27^, we report for the first time that the E2F transcription factors E2F4 and E2F5 play a role in regulating NIK transcription and expression. Overall, these data reveal critical roles for the regulation of NIK at the transcriptional level in propagating glioma cell invasion.

Utilizing specific cytokine treatments for preferential activation of the canonical or noncanonical NF-κB pathway by TWEAK or TNFα, respectively, we demonstrated that noncanonical NF-κB pathway activation increased invasion. Although we demonstrate that NIK promotes invasion in a noncanonical NF-κB-dependent manner, this does not exclude possible involvement of the canonical NF-κB pathway in regulating cancer invasion through independent or complementary mechanisms. Furthermore, we have previously demonstrated that NIK promotes mitochondrial fission and trafficking to the periphery of glioma cells during cell migration in a manner that is independent of IKKα/β and downstream NF-κB signaling^28^, suggesting that increased NIK transcription in lead invading cells may facilitate mitochondrial energy dynamics that support collective invasion.

A hallmark of invasion is alteration of the extracellular matrix to facilitate cell mobility, which includes changes in the dynamics of such proteins as e-cadherins, integrins or matrix metalloproteinases. In conjugation to promoting invasion, NIK has also been shown to regulate matrix metalloproteinase 14 (MMP14) with a reduction in phosphorylated MMP14, or its active form, seen in cells lacking NIK, where the opposite held true in cells expressing a constitutively active form of NIK^12^. RNA-seq analysis also revealed elevated MMP9 expression in glioma cells stimulated with TWEAK, which has been associated with cancer biomarkers^18^. Furthermore, as collective invasion maintains cell-to-cell contact through the extracellular matrix, it has been shown that stabilization of integrins is associated with increased collective invasion of solid tumors^29^, coinciding with an increase in integrin alpha 11 and integrin beta 3 gene expression in highly invading, TWEAK-treated cells. Integrin alpha 11 and integrin beta 3 have also been linked to increased tumor progression in other cancer types^19–21^. RNA-seq disease pathway analysis also highlighted TWEAK-treated glioma cells as having higher activation of cell-cell junctions and tumor development, invasion, and metastasis. Elevated matrix metalloprotease, integrin, and tumor progression is consistent with a leader phenotype displayed by NIK-expressing cells during collective invasion.

NIK and NF-κB dysregulation has been highly correlated with the induction of disease and malignancies^30–32^. Several studies have demonstrated an increase in NIK expression in various cancer models. Of these studies, NIK elevation was observed in breast cancer, lymphomas, pancreatic cancer, gastric cancer, and GBMs^11,33–36^. Given the pro-tumorigenic role NIK has in cancer and our studies suggesting that upregulation of NIK gene expression by cytokines in the tumor microenvironment can robustly trigger invasion, the inhibition of NIK may prove a promising therapeutic target for primary as well as recurrent or invasive/metastatic tumors^22,37–39^.

## Materials And Methods

### Reagents

TNFα (ProSpec CYT-223) and TWEAK (PeproTech 31006) were used at 10 ng/mL, mangiferin was obtained from Mangiferia indica leaves (M3547–100MG, Sigma Aldrich), collagen type I (354249) was purchased from Corning, and DiD and DiO were purchased from Invitrogen.

### Cell Lines

BT25, BT114 and BT116 cell lines were obtained from human GBM patients as previously described^40^. These cell lines were maintained as spheroids in neural stem cell medium containing DMEM/F-12, 1x B-27 supplement minus vitamin A, 1x GlutaMAX, 25 ng/mL EGF, 25 ng/mL basic fibroblast growth factor (bFGF), and 1x penicillin/streptomycin (Life Technologies).

### Plasmids

For NIK overexpression, mouse (mNIK) cDNA was cloned into pLenti6-V5-DEST (Addgene, Cambridge, MA) using the GATE-WAYTM Cloning System (Invitrogen). For pNIK-tagbroblastRFP, the promoter of NIK was purchased from Genecopoeia (HPRM17530) and cloned into a lentiviral plasmid.

### CRISPR-Cas9 gene knockout

BT25, BT114, and BT116 cells were transduced with a mixture of LentiCrispR-v2 carrying three gRNAs for each target. The gRNA sequences for human NIK E2F4 and E2F5 are shown in Supplemental Table 1.

### RNA Sequencing

1 × 10^6^ BT25 GBM cells harvested in untreated, TWEAK-treated (10 ng/mL for 4 hrs) and TNFα-treated (10 ng/mL for 30 minutes) conditions along with stable transgenic BT25 NIK KO cells. Duplicates of cell pellets were shipped on dry ice to Azenta by Genewiz (South Plainfield, NJ, USA), where RNA was isolated, and they conducted sequencing and constructed the sequencing library. For sequencing, HiSeq 2×150 bp was used. Sequence reads were trimmed using Trimmomatic v.0.36. The trimmed reads were mapped to the Homo sapiens GRCh38 reference genome using the STAR aligner v.2.5.2b. Unique gene hit counts were calculated by using featureCounts from the Subread package v.1.5.2. The hit counts were summarized and reported using the gene_id feature in the annotation le. Only unique reads that fell within exon regions were counted. DESeq2 les were uploaded to Ingenuity Pathway Analysis software (IPA; QIAGEN, Germantown, MD, USA) for dataset comparisons and to analyze altered genes that were statistically significant compared to wild type. IPA software was also used to analyze overall changes in disease function pathways related to identified gene families. GraphPad Prism (San Diego, CA, USA) was used to generate volcano plots and heatmaps.

### Immunoblot assays

Cells were lysed in RIPA lysis buffer (Pierce, #89900, Rockford, IL) with a protease/phosphatase inhibitor cocktail (Thermo Scientific). Equal amounts of protein were mixed with NuPage 4X LDS sample buffer (Invitrogen, NP0008) containing reducing agent and denatured at 100 °C for 7 min. Proteins were separated by 8–12% SDS-PAGE and transferred to nitrocellulose membranes (Bio-Rad, #162- 0115). The membranes were blocked for 1 h with 5% nonfat dry milk in 0.1% Tween-20/TBS (TBST) or Odyssey blocking buffer (LI-COR Biosciences, 927-40000) and incubated with primary antibodies from Cell Signaling Technology (CST) or Santa Cruz (SC): NIK CST4994, RelB CST4922, Lamin A CST 4777, NFKB2 CST4882, E2F3 SC56665, E2F4 SC69686 E2F5 SC374268, and Actin SC69879.

### Immuno fluorescence staining

Collagen-embedded spheroids were seeded on eight-well chamber slides (#80827, Ibidi, Munich, Germany) or 96 half-area well plates and allowed to adhere for 2 hours. During spheroid and monolayer invasion live cell imaging, cells were labeled with DiO or DiD for 30 minutes at 37°C before washing three times in media. Cells were allowed to invade for 48 to 72 hours, fixed with 4% paraformaldehyde, and permeabilized for 20 minutes with 0.3% Triton X-100 in PBS. Cells were incubated overnight in 0.1% Triton X-100 and 1% BSA in PBS at 4°C. Cells were then incubated in 1% BSA for 1 hour at room temperature. Cells were counterstained with the nuclear stain DAPI (Invitrogen, P36931).

### Three-dimensional collagen invasion assay

Monolayer invasion assays were performed as previously described [28]. Brie y, collagen type I (Corning, NY) was diluted to 2 mg/ml in DMEM/F-12 medium (1x Pen/Strep), and matrices were polymerized in 96-well plates. A total of 4 × 105 cells cultured in NSCs or NSCs+10% serum were seeded in triplicate in 100 μl DMEM/F-12 (1x Pen/Strep, 1x Glutamax) without growth factors or serum. Cells were fixed with 3% glutaraldehyde solution after 48 hours of invasion and stained with 0.1% toluidine blue. Invasion density was quantified by counting cells below the plane of the monolayer by bright-field light microscopy using a 10 × 10 ocular grid at 10x or 20x magnification corresponding to a 1 mm 2 field. Numbers in at least three equivalent, random fields were counted (n = 3 wells each) and normalized to the corresponding control. All experiments were performed at least three times.

Live cell invasion assays were performed using GBM cells. The cells were collected and centrifuged at 1.0 rcf for 2.5 minutes, and the medium was removed and disassociated with accutase for 9 minutes at room temperature before centrifugation at 1.0 rcf for 2.5 minutes. Accutase was removed, and cells were resuspended in NSC media and then quantified. Approximately 2.0×10^6^ cells were transferred into a 15 mL conical tube, and media was added to 2 mL with 12 μL of DiO added. Cells were incubated for 30 minutes at 37°C with DiO, followed by centrifugation at 1.0 rcf for 2 minutes. Cells were washed three times in NSC media before requantification. Cells were either used for monolayer invasion assay at 40,000 cells/well or 1.2×10^6^ cells were incubated for one week at 37°C in NSC media before being embedded into 2.0 mg/ml collagen matrix.

After spheroid formation, spheres were collected at 1.0 rcf for 2.0 minutes and resuspended in 2 mL of fresh media. The collagen matrix was prepared, approximately 60 μL of resuspended spheres was added to the matrix, and 18 μL of spheres embedded in collagen was added to each well. Collagen was allowed to solidify for 2 hours at 37°C before taking initial images. Images were acquired over a 72-hour time course.

### Image Acquisition

Images were acquired with a Nikon TI A1R inverted confocal microscope with a CFI60 Plan Apochromat Lambda 10x objective lens. Images were acquired with the following scan parameters: a “frame” scan mode of 1024 × 1024 pixels with a 16 bit depth and a grating of 3 rotations. Three-dimensional projections were obtained through Z stack images with 0.4700 μm between each image.

### RNA isolation, cDNA synthesis, and quantitative RT-qPCR

Total RNA was isolated from cells by a Purelink^™^ RNA Mini Kit (Life Technologies). cDNA was synthesized from 1 μg of total RNA using iScript reverse transcription supermix (Bio-Rad, Hercules, CA) following the manufacturer’s protocol. Quantitative RT-PCR was performed using iTaq Universal SYBR Green Supermix (Bio-Rad) with a StepOnePlus Real-Time PCR System (Applied Biosystems, Foster City, CA). The primers used are listed in Supplemental Table 2. The expression of mRNA was normalized to GAPDH expression levels. All experiments were performed at least three times with three replicates per sample.

## Data Availability

All data generated or analyzed during this study are included in this published article (and its Supplementary Information les).
